# The Role of Sex Hormones on Bone Mineral Density, Marrow Adiposity, and Muscle Adiposity in Middle-Aged and Older Men

**DOI:** 10.3389/fendo.2022.817418

**Published:** 2022-02-21

**Authors:** Li Xu, Qian Zhao, Kai Li, Yong Zhang, Chao Wang, Karen Hind, Ling Wang, Yandong Liu, Xiaoguang Cheng

**Affiliations:** ^1^ Department of Radiology, Beijing Jishuitan Hospital, Beijing, China; ^2^ International Medical Center, West China Hospital, Sichuan University, Chengdu, China; ^3^ Department of Epidemiology and Biostatistics, Beijing Research Institute of Traumatology and Orthopedics, Beijing Jishuitan Hospital, Beijing, China; ^4^ Department of Sport and Exercise Sciences, Durham University, Durham, United Kingdom

**Keywords:** bone mineral density, marrow adiposity, muscle adiposity, follicle-stimulating hormone, men

## Abstract

**Purpose:**

The etiology of age-related bone loss is less clear in men. This study is aimed to observe the variations of endogenous sex hormone concentrations with increasing of age in men, and investigate their relations to bone mass, marrow adiposity, and muscle adiposity.

**Methods:**

A total of 199 community-dwelling Chinese men (aged 41 to 82 years) were included and measured of serum total estradiol, total testosterone, and follicle-stimulating hormone (FSH) concentrations by enzyme-linked immunosorbent assay (ELISA). Vertebral trabecular volumetric bone mineral density (vBMD) was measured by quantitative computed tomography for all participants, and vertebral marrow fat content and erector muscle fat content were quantified by Chemistry-shift-encoding magnetic resonance imaging in 62 participants.

**Results:**

In this population, FSH concentration increased (*p* < 0.001) gradually with aging. Lower vBMD was independently associated with higher FSH concentration (β = -0.216, *p* < 0.001), but not with total estradiol or total testosterone. For each standard deviation increase in FSH there was a 50% higher risk of an individual having osteopenia or osteoporosis (vBMD < 120 mg/cm^3^). Marrow fat content and erector muscle fat content were greater in osteopenic and osteoporotic men, but there were no associations with sex hormones concentrations.

**Conclusion:**

In summary, FSH but not total estradiol or total testosterone is related to vertebral trabecular vBMD in middle-aged and older Chinese men. Neither marrow adiposity nor muscle adiposity is associated with sex hormones.

## Introduction

Although men do not experience a phase of accelerated bone loss similar to the menopause in women, their bone health status declines gradually with age (1). According to the World Health Organization (WHO) diagnostic standard using bone mineral density (BMD) measured by dual-energy X-ray absorptiometry (DXA), approximately 3-6% of men have osteoporosis and 28-47% have osteopenia, and the prevalence of osteoporosis rises to 19% in men who are aged 50 and older (2, 3). While it is established that the primary cause of menopausal-bone loss in women is driven by ovarian failure, in men, the etiology of age-related bone loss is less clear.

In men, sex steroids have an important role in the maintenance of bone strength, as demonstrated by the rapid reductions in bone density following androgen deprivation therapy (4). There is evidence of a small and progressive age-related decline in free testosterone, estradiol and to a lesser extent, progesterone (5, 6) and increases in luteinizing hormone, follicle-stimulating hormone (FSH) and sex hormone-binding globulin (SHBG) (7). However, research into the role of age-related sex hormone changes in the development of male osteoporosis has primarily focused on testosterone and estradiol. Endogenous testosterone levels have been related to bone turnover markers, BMD, and hip structural geometry in normal aging men (8–11). The actions of testosterone on the male skeleton are partly mediated by the aromatization of testosterone to estradiol, with estrogen deficiency also contributing to age-related bone loss in men, and there is cross-sectional and longitudinal evidence to suggest that bioavailable estradiol is more strongly associated with male BMD and fracture (10, 12, 13). Testosterone has been suggested to also play an indirect role in male skeletal health with aging by allowing for relative maintenance of balance and muscle strength in men compared to women (14). SHBG levels in men increase with age and influence the amount of hormone that is available to enter cells, and are predictive of vertebral fracture (15, 16) and/or lower bone density (17).

Relatively less attention has been directed toward the role of other sex steroids in male bone health, particularly of FSH. In women, a sharp rise in serum FSH during late perimenopause coincides with the most rapid rate of bone loss (18) and epidemiological data suggest that bone turnover markers and BMD in perimenopausal and early postmenopausal women are independent of serum estrogen, but negatively associated with FSH (19–21). However, the relationship between FSH and bone mass in men has not yet been fully explored.

Musculoskeletal fragility associated with sarcopenia (loss of muscle mass) and osteopenia (loss of bone mass) can result in fall and fracture. People are diagnosed with osteoporosis often combining with muscles weakness, and increased spine kyphosis leading vertebral fractures (22). Partial androgen deficiency may contribute to the age-related decrease in muscle mass and strength and to the higher risk of fall and fractures in elderly men (23, 24).

In the present cross-sectional study, we observed the variations of endogenous sex hormone concentrations with increasing of age, and investigated their relations to bone mass. Considering the potential roles of marrow adiposity and paravertebral muscle fragility in bone loss and vertebral fractures in old men which may be correlated with sex hormones, we furthermore explored the relationships between endogenous sex hormones concentrations and vertebral marrow fat content as well as paravertebral muscle fat content.

## Methodology

### Study Subjects

A total of 199 men aged from 41 and 82 years were recruited from a population study to investigate the degeneration of spine and knee (China Action on Spine and Hip study). All subjects were healthy adults who have lived in Beijing for more than 5 years, and the subjects with the following conditions were excluded: (1) spine or knee disorders due to congenital, tumor or tuberculosis; (2) a history of spine or knee injury or surgery; (3) suffering from other major diseases (such as infection, tumor, rheumatic immune disease, renal failure, coronary heart disease, stroke, and mental diseases) and taking bone metabolism regulating drugs; (4) heart pacemaker, coronary stent, orthopedic implants, and implant teeth; and (5) familial hereditary disease (25). The regional ethics committee approved the study and all participants provided signed, informed consent. The study was carried out in accordance with The Code of Ethics of the World Medical Association (Declaration of Helsinki).

All participants received blood serum tests for the measurement of total estradiol, total testosterone and FSH, and a quantitative computed tomography (QCT) scan for the measurement of vertebral trabecular volumetric BMD (vBMD). Sixty-two participants received Chemistry-shift-encoding magnetic resonance imaging (CSE-MRI) (mDixon-MRI) scans for vertebral marrow fat content and erector muscle fat content.

### QCT Scan and Measurements

We performed quantitative CT scan using a Toshiba CT scanner (Aquilion Prime ESX-302A; Toshiba Medical Systems Corporation, Otawara, Japan) with a five-rod calibration phantom (Model 3 phantom; Mindways Inc., Austin, TX, USA) placed beneath the subject and scanned simultaneously. The scanning parameters were as follows: 120kV, 187mAs, table height 120cm, 1 mm slice thickness, field of view (FOV) 500 mm. The scan range included 2 cm above L1 vertebra to 2 cm below L5 vertebra. Reconstruction parameters were standard algorithm, 1 mm section thickness and interval, and 400 mm display FOV.

The CT data were transferred to QCT workstation and analyzed using Mindways QCT PRO three-dimensional (3D) spine module software version 4.2. On the 3D reconstructed images, an elliptical cylinder region of interest (ROI) was individually placed in the central part of L2, L3, and L4 vertebral bodies. The ROIs contained the largest areas of the trabecular bone, but not included the cortical bone or basivertebral plexus. The values of vBMD of L2, L3, and L4 were automatically output. vBMD of lumbar spine was calculated as the mean value of vBMD of L2 to L4.

### CSE-MRI Scan and Measurements

On the same day as the QCT examination, the participants underwent a multiecho 3D spoiled gradient-echo sequence, referred to as an mDixon-Quant study, by using a 3.0-T MRI system with a 32-channel torso body coil (Ingenia, Philips Healthcare, Best, the Netherlands). The mDixon-Quant sequence is a 3D-FFE sequence, and uses multiple acquired echoes to generate water, fat, T2*, R2*, and in-phase and oppose-phase images synthesized from the water-fat images. The scan parameters of the single breath-hold mDixon-Quant were as follows: repetition time, 9.1 ms; echo time 1, 1.33 ms; six echoes with echo time shift, 1.3 ms; FOV, 360 × 330 × 120 mm^3^; flip angle, 3°; voxel size, 2.5 × 2.5 × 3.0 mm^3^; sensitivity encoding, 2; number of signal average, 2; and scan time, 12.5 s.

The CSE-MRI dataset were processed with ISP version 7 software (Philips Healthcare, Best, the Netherlands). ROIs for marrow fat content measurement were drawn manually encompassing the largest region of the cancellous bone of vertebral bodies on central L2, L3, and L4 axial image eliminating the vertebral cortex, schmorl’s nodules or hemangiomas. Marrow fat content was calculated as the mean value of L2, L3, and L4 marrow fat contents. The fat content of erector muscle was measured on the same central L3 image. Clear cavities of fat at the periphery of the erector muscle visually identify the edge of the muscle.

### Sex Hormone Analyses

For the assessment of endogenous hormone concentrations, a fasted, morning blood sample was taken from each participant on the same day as the imaging examinations. Blood was centrifuged, separated and refrigerated at -80°C until assay. Serum was sent to the laboratory at the Dopbio Biotechnology Co., Ltd for determination of testosterone, estradiol, and FSH concentrations.

Hormone assays were conducted by enzyme-linked immunosorbent assay (ELISA) using the MULTISKAN MK3 automated analyzer (Thermo Scientific, USA). Serum total estradiol concentrations were measured with a solid phase ELISA (DRG Estradiol ELISA, EIA-2693) based to the principle of competitive binding. Inter- and intra-assay coefficients of variation (CV) averaged 9.0% and 10.9%, respectively over the assay range. Serum FSH concentrations were measured with a two-step capture enzyme immunoassay test using constant amounts of two monoclonal antibodies (ALPCO FSH ELISA, 11-FSHHU-E01). One monoclonal antibody specific for FSH is immobilized onto the microplate and another monoclonal antibody specific for a different region of FSH is conjugated to horseradish peroxidase. Inter- and intra-assay CV averaged 4.2% and 5.2%, respectively. Total testosterone concentrations were determined with a competitive enzyme immunoassay (R&D Testosterone Parameter Assay Kit, KGE010). A monoclonal antibody specific for testosterone bounded to the goat anti-mouse antibody coated onto the microplate and then testosterone in a serum sample competed with a fixed amount of horseradish peroxidase-labeled testosterone for sites on the monoclonal antibody. Inter- and intra-assay CV averaged 3.3% and 6.2%, respectively.

### Statistical Analysis

Data management and analyses were completed using SPSS version 22.0 (SPSS Inc., Chicago, IL, USA). Distributions of the variables were examined for departure from normality by the Shapiro-Wilk test. Data with normal distribution are presented as the mean ± standard deviation (SD), while data with skewed distribution are presented as the median (interquartile, IQR). For the inter-group comparisons, *t* test was used for normally distributed variables and Mann-Whitney *U* test was used for skewed variables. Multivariate linear regression analysis was performed to estimate the contributions of each sex hormone to vBMD, marrow fat content, and erector muscle fat content, with and without adjustments for age, BMI, and other sex hormones. According to the criteria suggested by the International Society for Clinical Densitometry in 2007 (26) and by the American College of Radiology in 2008 (27) for QCT, the thresholds of vBMD were < 120 mg/cm3 for osteopenia and < 80 mg/cm3 for osteoporosis. The odds ratios (ORs) and 95% confidence intervals (95% CI) of osteopenia/osteoporosis (defined as vBMD < 120 mg/cm^3^), higher marrow fat content (marrow fat content ≥ 45%), and higher muscle fat content (erector muscle fat content ≥ 10%) were calculated by logistic regression models with and without adjustments for age, BMI, and other sex hormones, in which sex hormone levels were fitted as continuous variables with results expressed in terms of per SD increase in hormone concentration.

## Results

The general characteristics of the study participants are shown in **Table 1**. Participants were stratified into four sub-groups according to age, and the characteristics of each group and inter-group comparisons are also presented in **Table 1**. FSH concentrations were higher with increasing age (*p* < 0.001), but there were no age related differences in total estradiol concentration (*p* = 0.90). With aging, lumbar spine vertebral vBMD decreased (*p* < 0.001) and erector muscle fat content increased (*p* = 0.034) gradually. There were no significant age-related differences in marrow fat content (*p* = 0.574).

**Table 1:** Demographic and biologic characteristics of 199 men overall, and of the men at different levels of age.

	Mean Value ( ± SD)/Median (IQR)^b^					
	All	age ≤ 50 yrs	50 < age ≤ 60 yrs	60 < age ≤ 70 yrs	age > 70 yrs	*P* value^c^
	(n = 199)	(n = 35)	(n = 51)	(n = 75)	(n = 38)	
**Age (years)**	61	46	56	64	72	<0.001
	(52, 68)	(44, 47)	(52, 58)	(61, 67)	(71, 75)	
**BMI (kg/m^2^)**	24.8	23.1	25.4	25.6	24.85 ± 4.14	0.062
	(22.4, 28.9)	(21.7, 25.3)	(22.8, 29.8)	(22.6, 34.4)		
**Follicle-stimulating hormone (IU/L)**	10.87	9.02 ± 3.60	10.70	11.09	13.42	<0.001
	(7.52, 16.55)		(6.42, 16.48)	(7.67, 16.39)	(9.03, 26.39)	
**Estradiol (pg/mL)**	32.66	30.24	34.94	30.05	39.10	0.90
	(19.62, 51.24)	(15.97, 52.37)	(19.84, 53.14)	(20.58, 50.44)	(19.31, 46.83)	
**Testosterone (ng/mL)**	7.90	5.50	8.55 ± 4.16	9.54 ± 4.69	7.20	0.017
	(4.98, 11.18)	(3.50, 8.33)			(4.82, 9.68)	
**Volumetric bone mineral** **density (mg/mm^3^)**	115.07 ± 32.51	139.07 ± 24.11	122.69 ± 23.49	111.64 ± 32.31	89.52 ± 31.15	<0.001
**Marrow Fat Content (%)** ^a^	44.67 ± 8.23	39.84 ± 6.97	44.90 ± 7.28	45.09 ± 9.28	46.91	0.574
					(45.16, 47.06)	
**Erector Muscle Fat Content (%)** ^a^	8.44 ± 4.63	5.57	7.74 ± 3.62	8.46 ± 4.25	12.67 ± 6.97	0.034
		(3.04, 5.57)				

a For marrow fat content and erector muscle fat content measurements, number of the whole study sample group and of each group stratified by age with an interval of 10 years was as follow: 62 for all, 5 for the group aged no more than 50 years, 15 for the group aged between 50 to 60 years, 35 for the group aged between 60 to 70 years, and 7 for the group aged over 70 years, respectively;

b Continuous variables were tested by the Shapiro-Wilk test: normally distributed variables are presented as mean± SD, while skewed variables are presented as the median (first-fourth quartiles);

c For the comparisons among the groups stratified by age, t test was used for normally distributed variables and Mann-Whitney U test was used for skewed variables.

Further sub-group analysis was completed according to presence of osteopenia and osteoporosis (normal bone mass: vBMD ≥ 120 mg/mm^3^ and osteopenia/osteoporosis: vBMD < 120 mg/mm^3^) and of obesity (non-obesity: BMI < 25 kg/m^2^ and obesity: BMI ≥ 25 kg/m^2^), respectively, and inter-group comparisons are shown in **Table 2**. Participants who were osteopenic or osteoporotic had significantly higher FSH concentrations (*p* = 0.005), marrow fat content (*p* = 0.002), and erector muscle fat content (*p* = 0.003). Obese participants had significantly higher total testosterone concentrations (*p* = 0.004), but there were no differences in vBMD or fat content measurements.

**Table 2:** Comparisons of demographic and biologic characteristics between the participants with normal bone mineral content and with decreased bone mineral content (osteopenia or osteoporosis), and between non-obese and obese participants.

		Mean Value ( ± SD)/Median (IQR)^b^						
	Normal bone mass (vBMD ≥ 120 mg/mm^3^) (n = 92)	Osteopenia/Osteoporosis (vBMD < 120 mg/mm^3^) (n = 107)	*P* value^c^	*power*	BMI < 25 kg/m^2^	BMI ≥ 25 kg/m^2^	*P* value^c^	*power*
					(n = 105)	(n = 94)		
**Age (years)**	58	65	<0.001	1	60	61	0.227	0.301
	(47, 61)	(58, 70)			(50, 69)	(57, 67)		
**BMI (kg/m^2^)**	24.4	25.1	0.410	0.220	22.2	29.2	<0.001	1
	(22.4, 27.5)	(22.3, 30.0)			(20.8, 23.7)	(26.3, 36.8)		
**Follicle-stimulating hormone (IU/L)**	9.35	11.45	0.005	0.933	10.95	10.80	0.565	0.088
	(6.94, 13.7)	(7.97, 19.4)			(7.67, 17.15)	(7.10, 16.23)		
**Estradiol (pg/mL)**	31.8	34.19	0.615	0.131	34.70	29.99	0.619	0.064
	(19.43, 52.95)	(19.62, 49.72)			(18.06, 52.38)	(20.50, 48.53)		
**Testosterone (ng/mL)**	7.81	7.97	0.933	0.052	6.61	9.17	0.004	0.720
	(4.98, 10.85)	(4.85, 11.38)			(4.73, 9.68)	(5.33, 12.38)		
**Volumetric bone mineral density (mg/mm^3^)**	138.42	94.17	< 0.001	1	117.73 ± 34.40	112.11 ± 30.17	0.341	0.024
	(128.38, 155.42)	(77.86, 107.89)						
**Marrow Fat Content (%)** ^a^	40.74 ± 7.83	47.16 ± 7.56	0.002	0.838	42.08	44.49 ± 8.27	0.938	0.062
					(39.78, 52.10)			
**Erector Muscle Fat Content (%)** ^a^	6.31 ± 4.11	9.79 ± 4.47	0.003	0.843	8.79 ± 5.46	8.35 ± 4.44	0.685	0.299

a For marrow fat content and erector muscle fat content measurements, number of those with normal bone mineral content, those with decreased bone mineral content (osteopenia/osteoporosis), those of non-obesity (BMI < 25), and those of obesity (BMI ≥ 25) was 24, 38, 13, and 49, respectively;

b Continuous variables were tested by the Shapiro-Wilk test: normally distributed variables are presented as mean± SD, while skewed variables are presented as the median (first-fourth quartiles);

c For the comparison between normal bone mineral content and osteopenia/osteoporosis and the comparison between non-obesity (BMI < 25) and obesity (BMI ≥ 25), t test was used for normally distributed variables and Mann-Whitney U test was used for skewed variables.

Associations between sex hormone concentrations, vBMD, and fat content measurements are shown in **Table 3**. Lower vBMD was associated with higher FSH concentration (β = -0.216, *p* < 0.001), but not with total estradiol or total testosterone concentration (**Table 3**), after adjustment for age, BMI, and other sex hormones. Neither bone marrow fat content nor erector muscle fat content was associated with sex hormone concentrations after adjustment for age, BMI, and other sex hormones.

**Table 3:** Multivariate linear regression analysis showing the factors determining vBMD and fat measurements.

	Follicle-stimulating hormone		Estradiol		Testosterone	
	Unadjusted	MV adjusted^a^	Unadjusted	MV Adjusted^a^	Unadjusted	MV Adjusted^a^
	β	β	β	β	β	β
**Volumetric bone mineral density**	-0.349^**^	-0.216^**^	0.017	0.014	-0.027	-0.018
**Marrow Fat Content**	0.181	0.164	-0.087	-0.099	-0.011	0.052
**Erector Muscle Fat Content**	0.114	-0.006	-0.140	-0.119	-0.248^*^	-0.145

a Adjusted for age, BMI and mutual adjustment for other sex hormones;

^*^Indicates 0.01 ≤ p < 0.05, and ^**^indicates p < 0.01.

Adjusted and non-adjusted logistic regression analysis found that for each SD increase in FSH there was a 50% higher risk of an individual having osteopenia or osteoporosis (**Table 4**). All the three sex hormones showed no significant contributions to the risk of higher marrow fat content or higher erector fat content after multivariate adjustment.

**Table 4:** OR of osteopenia/osteoporosis, higher marrow fat, and higher muscle fat (erector muscle fat content ≥ 10%) per SD increase in FSH, estradiol, and testosterone concentrations in men.

	Follicle-stimulating hormone		Estradiol		Testosterone	
	Unadjusted OR (95% CI)	MV adjusted^a^ OR (95% CI)	Unadjusted OR (95% CI)	MV adjusted^a^ OR (95% CI)	Unadjusted OR (95% CI)	MV adjusted^a^ OR (95% CI)
**Osteopenia/Osteoporosis**	1.895^**^	1.509^*^	0.886	0.853	0.983	0.881
	(1.299-2.762)	(1.015-2.242)	(0.669-1.172)	(0.618-1.179)	(0.743-1.299)	(0.629-1.234)
**higher marrow fat content (≥ 45%)**	1.374	1.186	0.867	0.801	0.580	0.702
	(0.859-2.199)	(0.695-2.024)	(0.485-1.550)	(0.407-1.575)	(0.289-1.166)	(0.332-1.484)
**higher erector muscle fat content (≥ 10%)**	1.124	0.994	0.803	0.715	0.475	0.524
	(0.717-1.761)	(0.561-1.760)	(0.421-1.529)	(0.332-1.538)	(0.215-1.051)	(0.220-1.247)

a Adjusted for age, BMI and mutual adjustment for other sex hormones.

^*^Indicates 0.01 ≤ p < 0.05, and ^**^indicates p < 0.01.

## Discussion

This study is the first to explore associations between sex hormones, bone density, marrow adiposity and muscle adiposity in community-dwelling middle and older aged men. In doing so, we found that high concentration of FSH, but not total testosterone or estradiol, was a risk factor for lower vBMD, osteopenia and osteoporosis. While marrow fat content and erector muscle fat content were greater in osteopenic and osteoporotic men, there were no associations with sex hormones concentrations.

Our primary finding was of significant associations between high FSH and low vertebral trabecular vBMD in men. There is accumulating evidence that FSH acts on bone through both direct and indirect mechanisms. First, FSH acts directly through the FSHR on osteoclast precursors to increase osteoclastogenesis by sensitizing MAP kinase, NF-*κ*B, and Akt pathways, and blocking FSHRs or the β-subunit of FSH, can prevent bone loss in mice independent of estrogen (28). Second, FSH increases expression of the receptor activator of NF-κB (29) and indirectly stimulates osteoclastogenesis by releasing cytokines, namely IL-1*β*, TNF-*α*, and IL-6 in proportion to the surface expression of FSHR (21, 30). Elsewhere, *in-vivo* evidence regarding the relationship between FSH and bone mass in men is limited. Hsu B, et al. reported that lower levels of serum FSH were protective against bone loss and were negatively associated with incident fractures in a cohort of elderly men (31). In addition, Jing, et al. have demonstrated associations between FSH, lumbar spine BMD and osteoporosis in men with type 2 diabetes mellitus (32).

In this study, we found no associations between total estradiol, total testosterone and vertebral trabecular vBMD. Elsewhere, studies have supported estradiol as the crucial sex steroid for bone health in men, and others, provide evidence for testosterone and SHBG (8–11, 32–37). In elderly men, bioavailable estradiol has been found to be positively associated with BMD at multiple sites (8, 10, 11, 33–35), and higher bioavailable estradiol is suggested to be a protective factor for osteopenia (9). Total estradiol has also been reported to be a significant determinant of bone mass in elderly men (10, 24, 34, 36). In a study involving a diverse sample of men (Black, Hispanic, and White, aged 30 to 79 years), total and free estradiol showed larger positive correlations with BMD outcomes and correlations between estradiol levels and hip BMD were robust after multivariate-adjustment, whereas no correlation between total or free estradiol and lumbar spine BMD after multivariate-adjustment (8). In aging Chinese men, both bioavailable and total estradiol have been demonstrated with a positive association with both total hip and lumbar spine BMD after multivariate- or age-adjustment (32, 37). The bioavailable sex steroids comprise the fractions that are free or associated with albumin in the circulation (38), and it is these fractions that have rapid access to target tissues (39). The free fraction constitutes only 1-3% of the total circulating sex steroids (38). In contrast, the fraction bound to SHBG does not have free access to target tissue. As SHBG levels increase with age in men, measurement of total testosterone or estradiol levels does not accurately reflect the actual levels of these steroids available to tissue.

The evidence around the role of testosterone for bone health in elderly men is more conflicting, than the evidence for estradiol (9–11, 24, 34, 36). A study on American men aged 20–90 years found that men with free testosterone level at the lowest quartile were more likely to be osteopenic (9). A study by Van Den Beld et al. also found a positive and significant association between testosterone levels (total, free, and bioavailable fraction and androgen index) and proximal femoral BMD in older men (10). The Swedish Osteoporotic Fractures in Men (MrOS) Study discovered that free testosterone was an independent positive predictor of BMD in total body, total hip, femur trochanter, and arm but not in the lumbar spine (11). The Hong Kong MrOS study also found a positive and significant relationship between femoral and vertebral BMD and free testosterone in Chinese men aged 65 years and above (37). However, discrepancies exist among different studies, and some have reported no associations between bone health status in men and total testosterone (8, 9, 34), and studies have found that the relationship between testosterone and BMD is not consistent at different testing sites (35). The effects of testosterone can be exerted either directly through the androgen receptor or indirectly through aromatization to estrogens and further through estrogen receptor-α and/or –β (40). These sex steroid receptors are expressed in bone, and experimental animal studies, using sex steroid receptor-inactivated transgenic mouse model, have indicated that each of these three receptors mediate the site-specific skeletal effects of sex steroids (41, 42).

To date, most studies have been conducted using DXA measured BMD, which cannot separate trabecular from cortical bone. Trabecular bone is known to have a more rapid rate of age-related loss than cortical bone. This may diminish the sensitivity of DXA for assessing osteoporosis. QCT is a truly three dimensional technique for quantifying volumetric trabecular bone density that is not affected by spine degeneration and abdominal aortic calcification (43). Khosla et al, measured vBMD using QCT and showed that vertebral trabecular vBMD was significantly associated with bioavailable estradiol and bioavailable testosterone in the entire group of men (22 to 91 years) (33). After adjusting for age, bioavailable testosterone was more strongly associated with vertebral trabecular vBMD than bioavailable estradiol in the middle-aged men (40-59 years), while bioavailable estradiol was the best predictor of vBMD at most sites in the elderly men (≥ 60 years) (33).

In the current study, there were no associations between sex hormone concentrations and marrow fat content, despite evidence that marrow fat increases with age (44, 45). Mistry et al, have reported that marrow adiposity is negatively associated with both total estradiol and total testosterone in aging men (74 to 95 years of age) after adjusting for age and total percent fat (46). The possible reasons for the discrepancies with Mistry’s results may be firstly, the number size of this study (n = 62) was small and secondly, the participants in this study are much younger than those in Mistry’s study (61 years vs. 82.4 years). FSH receptor cDNA and protein have been identified on osteoclasts and mesenchymal stem cells (28). An animal study showed that both sham-operated and ovariectomized mice presented a decrease in marrow adipose volume after FSH antibody treatments (47). Our study did not find a significant association between marrow fat content and FSH concentration in this middle-aged to older male population, and furthermore, the increase of FSH was not a risk factor for higher marrow fat content.

In the context of muscle aging, it is important to remember that it is not just a decline in muscle mass which contributes to the deterioration of muscle function. Factors underpinning muscle quality come into play, including muscle composition, aerobic capacity and metabolism, fatty infiltration, insulin resistance, fibrosis and neural activation (48). Observational studies have shown that lean mass and strength are reduced in men with low testosterone levels (10, 23), and testosterone replacement increases lean mass in men with hypogonadism (49). In the present cross-sectional study, total testosterone concentration was detected with a weakly negative correlation with erector muscle fat content by unadjusted linear regression analysis, however, the correlation was insignificant after adjusting for age, BMI, total estradiol concentration, and FSH concentration. The increase of total testosterone for 1 SD was not a significant protective factor for higher erector muscle fat content (≥ 10%). Considering lean mass, muscle area, and muscle strength were preserved until gonadal steroid deficiency was more mark according to Finkelstein et al. (a testosterone level ≤ 200 ng per deciliter) (50), our results are not unexpected.

The main limitation of this study was the relative small sample size (n = 199), especially only 62 participants had MR measurements of marrow fat content and muscle fat content, and as a result, the relationships between sex hormones and MR-quantified marrow fat and muscle fat content may not be fully revealed. In addition, luteinizing hormone and SHBG were not measured, and therefore, free estradiol and free testosterone could not be calculated and investigated in this study. The smoking and drinking history were not obtained in this study, and therefore, neither multivariate linear regression analyses for the contributions of sex hormones to vBMD nor ORs of osteopenia/osteoporosis were adjusted for smoking and drinking.

## Conclusion

FSH but not total estradiol or total testosterone is related to vertebral trabecular vBMD in middle-aged and older Chinese men. Neither marrow adiposity nor muscle adiposity is associated with total estradiol, total testosterone, or FSH.

## Data Availability Statement

The original contributions presented in the study are included in the article/supplementary files. Further inquiries can be directed to the corresponding author.

## Ethics Statement

The studies involving human participants were reviewed and approved by Beijing Jishuitan Hospital Ethics Committee. The patients/participants provided their written informed consent to participate in this study.

## Author Contributions

The first authorship of LX and QZ is of an equal rank. LX and QZ designed the study and prepared the first draft of the paper. KL, YZ, and YL contributed the experimental work and data collection. KH and LW edited the draft. CW was responsible of the statistical analysis of the data. XC supervised the study and paper organization. All authors revised the paper critically for intellectual content and approved the final version. All authors agree to be accountable for the work and to ensure that any questions relating to the accuracy and integrity of the paper are investigated and properly resolved.

## Funding

This work was funded by the grants from the National Key Research and Development Program of China [No. 2020YFC2004902], the Beijing Hospitals Authority Clinical Medicine Development of Special Funding Support [No. ZYLX202107], the National Natural Science Foundation of China [No. 81771831, 2018], and the High Level Talents “Discipline backbone” Project of Beijing Jishuitan Hospital [No. XKGG2021123].

## Conflict of Interest

The authors declare that the research was conducted in the absence of any commercial or financial relationships that could be construed as a potential conflict of interest.

## Publisher’s Note

All claims expressed in this article are solely those of the authors and do not necessarily represent those of their affiliated organizations, or those of the publisher, the editors and the reviewers. Any product that may be evaluated in this article, or claim that may be made by its manufacturer, is not guaranteed or endorsed by the publisher.
